# Radiotherapy prolongs the survival of advanced non-small-cell lung cancer patients undergone to an immune-modulating treatment with dose-fractioned cisplatin and metronomic etoposide and bevacizumab (mPEBev)

**DOI:** 10.18632/oncotarget.20411

**Published:** 2017-08-24

**Authors:** Pierpaolo Pastina, Valerio Nardone, Cirino Botta, Stefania Croci, Paolo Tini, Giuseppe Battaglia, Veronica Ricci, Maria Grazia Cusi, Claudia Gandolfo, Gabriella Misso, Silvia Zappavigna, Michele Caraglia, Antonio Giordano, Donatella Aldinucci, Pierfrancesco Tassone, Pierosandro Tagliaferri, Luigi Pirtoli, Pierpaolo Correale

**Affiliations:** ^1^ Radiotherapy Unit, Department of Medicine, Surgery, and Neuroscience, Siena University Hospital, Siena, Italy; ^2^ Medical Oncology Unit, AUO “Mater Domini”, “Magna Graecia” University, Catanzaro, Italy; ^3^ Radiology Unit,Department of Medicine, Surgery, and Neuroscience, Siena University Hospital, Siena, Italy; ^4^ Department of Medical Biotechnology, Microbiology and Virology Unit, University of Siena, Siena, Italy; ^5^ Department of Biochemistry, Biophysics and General Pathology, University of Campania “L. Vanvitelli”, Naples, Italy; ^6^ Sbarro Institute for Cancer Research and Molecular Medicine, Center for Biotechnology, Temple University, Philadelphia, PA, USA; ^7^ Department of Medicine, Surgery, and Neuroscience, University of Siena and Istituto Toscano Tumori (ITT), Siena, Italy; ^8^ Department of Experimental Oncology 2, CRO Aviano National Cancer Institute, Aviano, Italy; ^9^ Medical Oncology Unit, Metropolitan Hospital “Bianchi-Melacrino-Morelli, Reggio Calabria, Italy

**Keywords:** immune-modulation, radiation therapy, metronomic chemotherapy, NSCLC, retrospective analysis

## Abstract

Radiotherapy (RT), together with a direct cytolytic effect on tumor tissue, also elicits systemic immunological events, which sometimes result in the regression of distant metastases (abscopal effect). We have shown the safety and anti-tumor activity of a novel metronomic chemotherapy (mCH) regimen with dose-fractioned cisplatin, oral etoposide and bevacizumab, a mAb against the vasculo-endothelial-growth-factor (mPEBev regimen), in metastatic non-small-cell-lung cancer (mNSCLC). This regimen, designed on the results of translational studies, showed immune-modulating effects that could trigger and empower the immunological effects associated with tumor irradiation. In order to assess this, we carried out a retrospective analysis in a subset of 69 consecutive patients who received the mPEBev regimen within the BEVA2007 trial. Forty-five of these patients, also received palliative RT of one or more metastatic sites. Statistical analysis (a Log-rank test) revealed a much longer median survival in the group of patients who received RT [mCH *vs* mCH + RT: 12.1 +/-2.5 (95%CI 3.35-8.6) *vs* 22.12 +/-4.3 (95%CI 11.9-26.087) months; *P*=0.015] with no difference in progression-free survival. In particular, their survival correlated with the mPEBev regimen ability to induce the percentage of activated dendritic cells (DCs) (CD3-CD11b+CD15-CD83+CD80+) [Fold to baseline value (FBV) ≤1 *vs* >1: 4+/-5.389 (95%CI,0- 14.56) *vs* 56+/-23.05 (95%CI,10.8-101.2) months; *P*:0.049)] and central-memory- T-cells (CD3+CD8+CD45RA-CCR7+) [FBV ≤ 1 *vs* >1: 8+/-5.96 (95%CI,0-19.68) *vs* 31+/-12.3 (95%CI,6.94-55.1) months; *P*:0.045].

These results suggest that tumor irradiation may prolong the survival of NSCLC patients undergone mPEBev regimen presumably by eliciting an immune-mediated effect and provide the rationale for further perspective clinical studies.

## INTRODUCTION

Non-small cell lung cancer (NSCLC) is the most common malignancy and the leading cause of cancer death, representing 17% of new cases of cancer diagnosed worldwide [1, 2]. Chemotherapy with platinum derivatives in combination with a second cytotoxic drug (gemcitabine, pemetrexed or taxans) is recommended for patients in advanced stage of disease (stage IIIB-IV) and no driver mutations/rearrangements, which, on the other hand, require molecular targeted specific drugs against EGFR or EML-ALK [3–5]. Further on, the addiction of Bevacizumab, an anti-angiogenic mAb that targets the vasculo-endothelial-growth-factor (VEGF), to the chemotherapy is recommended in patients with non-squamous histology being able to prolong their progression-free (PFS) and overall survival (OS). On the overall, PFS and OS of advanced NSCLC patients are in a range of 7-8 and 12-13 months, respectively. More recently, the clinical development of immune-check point blockade offers new treatment opportunities for these patients. In fact, mAbs to programmed cell death receptor-1 (PD-1) and PD-1 ligand-1 (PDL-1) may improve their survival by rescuing pre-existing tumor-specific cytotoxic-T-cells (CTLs) in the tumor sites [6–11]. Tumor specific-CTLs may rise in NSCLC patients in response to the processing and cross-priming of antigenic material spontaneously released in a context of an immunogenic danger signal by the tumor cells or as consequence of previous antitumor treatments, including CH and/or RT. The latter strategy in particular, alone or in combination with CH, represents a powerful palliative treatment in advanced NSCLC patients [12–17]. Most recent technological developments, including intensity/volumetric modulated arc therapy (IMRT/VMAT), image-guided RT (IGRT), 4D-conformal RT simulation and proton therapy, have greatly reduced the occurrence of adverse events, and its use is achieving excellent palliative results in term of symptom relief and in term of symptom relief and quality of life in patients with life-threatening metastases, mostly to bone, intra-thoracic and central nervous system lesions, and more rarely to the testicles [18–26]. Although RT is generally considered as a loco-regional anticancer means, a number of studies have largely demonstrated its ability to elicit complex immune-adjuvant effects. RT may, in fact, induce immunogenic cell death and peri-tumoral inflammation, thus converting the irradiated tumor tissue in a functional “*in situ*” antitumor vaccine with consequent poly-antigen specific CTL responses [27–29]. At this purpose, these immune-biological effects have been advocated to explain the sporadic occurrence of tumor regressions outside the irradiation site in cancer patients undergone palliative RT with no other concomitant systemic anticancer treatments [30–33]. This phenomenon recognized in the 50ths by Mule et al. was designated as “abscopal effect” [30–33]. On these bases, several trials are currently testing RT in combination with immunotherapy and/or immune-checkpoint blockade in patients with a number of different malignancies [28, 30, 34, 35] including malignant melanoma [34], prostate adenocarcinoma [35], and NSCLC. Several cytotoxic drugs and CH schedules have shown immune-modulating effects similar to RT. Metronomic chemotherapy (mCH) or dose-dense CH, is an alternative anti-cancer strategy based on the use of conventional cytotoxic drugs administered at lower dosage for a prolonged period of time [36]. This treatment modality allows the achievement of a higher dose-intensity of target cytotoxic drugs compared to traditional usage, avoiding dangerous peaks in blood concentration and, consequently, showing a more tolerated toxicological profile and different antitumor activity. The antitumor activity of mCH derives by a combination of different mechanisms concerning: a direct cytotoxic effect on tumor cells (anti-blastic effect), pro-angiogenic precursors (anti-angiogenic effect), immunosuppressive regulatory-T-cells (Tregs) and myeloid-derivative-suppressor-cells (MDSCs) (immune-modulating effects). Additionally, it may induce neo-antigen and gene modulation in tumor cells (epigenetic effect) and constant release of tumor-derived antigens in a context of immunogenic cell death (immune-stimulating effects) [36, 37]. We previously showed that a metronomic regimen with dose-fractioned cisplatin, oral etoposide and bevacizumab (mPEBev) is a safe and very active treatment for advanced NSCLC patients enrolled in the two-step phase I-IIb BEVA2007 trial [38–41]. An immune-biological monitoring performed on these patients along the treatment, revealed its ability to decrease the serum levels of multiple proangiogenic factors (VEGFA, Angiopoietin 2 and Follistatin), and cytokines (IFN0263, IL4 and IL17A). This treatment was also associated to a progressive increase of different circulating immune-cell lineages including activated CTLs (CD3+CD8+CD62L+), long-term effector-memory- (CD3+CD8+CD27+) and central-memory-T-cells (TCMs; CD3+CD8+C45RA-CCR7+). The BEVA2007 trial was enforced by a functional study on *in vitro* cultured peripheral blood mononuclear cells (PBMCs) derived from the enrolled patients. The results of this ancillary study revealed the ability of the mPEBev regimen to give rise to an efficient DC activity in the cultures and to promote an increased antigen-specific T-cell proliferation and Th1 cytotoxic response in NSCLC patients [39–41]. We have, therefore, hypothesized that the immune-modulating properties of the mPEBev regimen could empower the systemic immunological effect potentially ignited by conventional RT. In order to test this hypothesis, we carried out a retrospective analysis aimed to investigate whether the use of RT, given on palliative setting, could improve the outcome of NSCLC patients who received the mPEBev mCH regimen.

## RESULTS

### Study design

We carried out a retrospective analysis on sixty-nine consecutive patients with metastatic non-squamous NSCLC enrolled in the step 2 of the BEVA2007 trial between September 2007 and September 2015. Patients who completed four treatment cycles according to the mPEBev regimen were selected for our retrospective study. Among these, twenty-four patients received mCH alone and their features are presented in the Table 1. The remaining forty-five patients, 37 males and 8 females, with median age of 63 years and a median ECOG performance status of 1, presented symptomatic lesions and required concurrent palliative RT on selected sites (Table 1). Nineteen of the latter patients received RT to the bones; seven to thoracic lesions (five lung and two nodes); twenty-one to brain (eighteen WBRT and three SRS). Sixteen patients with bone metastases received 30 Gy in ten RT fractions, while three patients received further 20 Gy in five fractions. The treatment resulted safe in patients with advanced NSCLC undergoing to mCH and RT. Thirty Gy in ten RT fractions were planned for patients who received WBRT; while 54 Gy in 30 RT fractions were planned for patients who needed thoracic irradiation. Brain SRS was instead administered as 20 Gy in one single fraction. No significant adverse events or toxicity-related interruptions were recorded for these patients, and all of them could complete the pre-planned treatment program. However, there was a moderate hematological toxicity in both groups, mainly consisting in reversible grade 1-3 leucopenia (6 cases in the mCH group *vs* 8 cases in the mCH plus RT group) rapidly recovered with the use of growth factors, grade 2 anemia (6 cases in the mCH group *vs* 8 cases in the mCH plus RT group). In addition, only a light-moderate grade of skin toxicity and a grade 3 post-attinic esophagitis (1 case) were observed in the group of patients undergoing mCH plus RT. Palliative RT was administered after two and four mPEBev courses in 10 and 35 patients, respectively.

**Table 1:** Clinical features and treatment of sixty-nine patients (pts) enrolled in the BEVA2007 trial

Features	Patients who received palliative RT (45 patients)	Patients who did not receive palliative RT (24 patients)
Sex		
Male	37 (82.2%)	16 (66.7%)
Female	8 (17.8%)	8 (33.3%)
**RT sites**		
^1^Bone	19 (42.2%)	NA
^2^Lung	5 (11.1%)	NA
^3^Nodes	2 (4.4%)	NA
^4^WBRT	18 (40%)	NA
^5^SRS brain	3 (6.7%)	NA
Histology		
Adenocarcinoma	31 (68.9%)	16 (66.7%)
Squamous	7 (15.6%)	2 (8.3%)
Large cell carcinoma	1 (0.2%)	4 (16.7%)
NAS	6 (13.3%)	2 (8.3%)

Patients received four mCH courses according to the mPEBev regimen, and did not show progression of disease along the treatment. Forty-five of these patients received additional palliative radiotherapy (RT) on quality of life threatening lesions.

Radiotherapy Treatment and dosage: ^1^Thirty Gy in ten RT fractions (three patients received additional treatment with 20 Gy in five fractions); ^2-3^Fifty-four Gy in 30 RT fractions; ^4^Thirty Gy in ten RT fractions; ^5^Twenty Gy in one single fraction.

### Radiotherapy and survival

**Figure 1:**
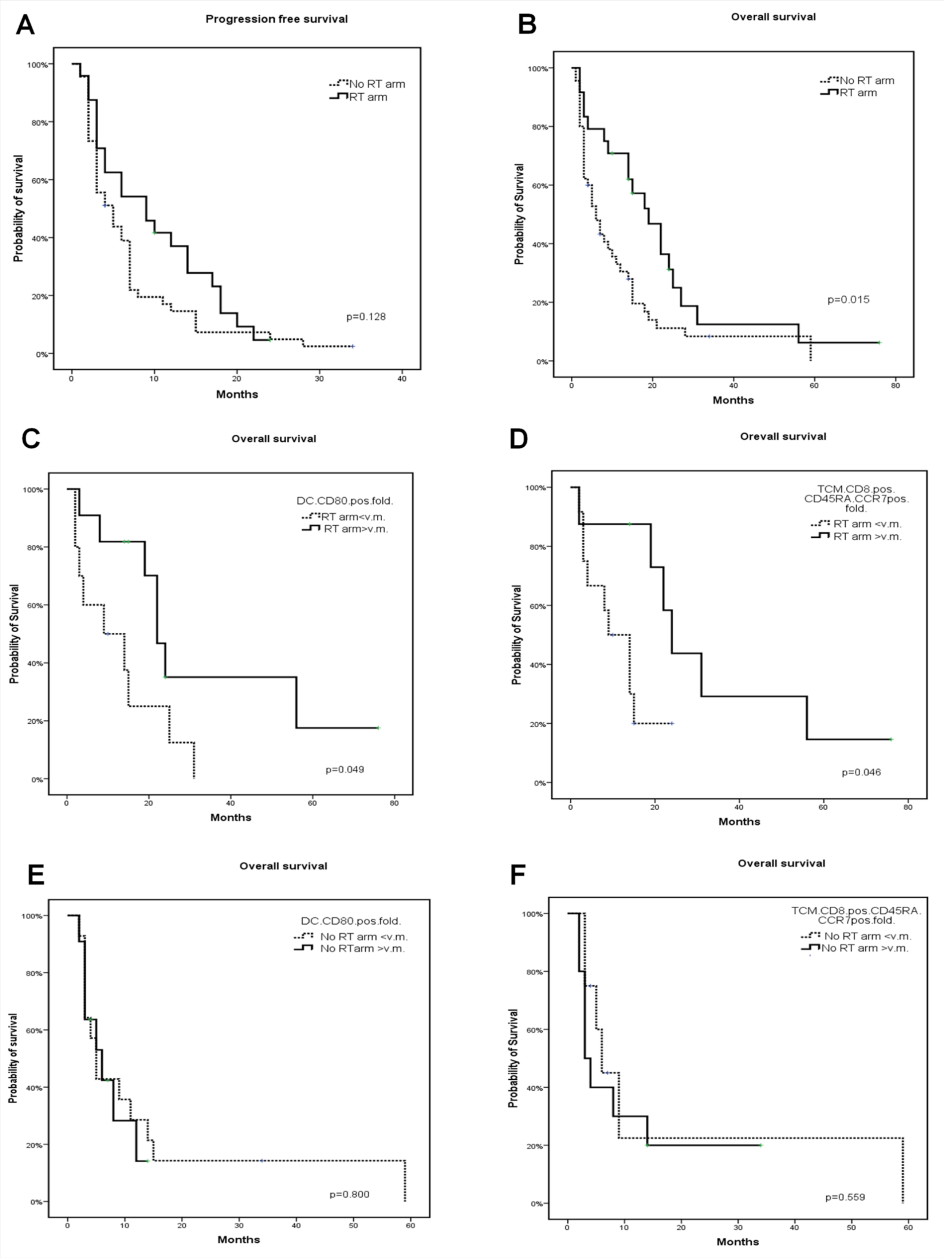
Representation of PFS and OS of patients PFS **A.** and OS **B.** of patients undergone mCH and mCH + RT. Figure also represents OS of patients undergone mCH **C.**, **D.** and mCH + RT **E.**, **F.** with fold to baseline value of activated dendritic cells **C.** and **E.** and central memory T cells **D.** and **F.** ≤ 1 or > 1. The percentage of activated dendritic cells and central memory T cells was evaluated on the PBMCs isolated from patients at baseline and after four mCH courses, by performing a multicolor immune-cytoflurimetric analysis, as described in a previous study on the same patients.

Kaplan-Meier curves and log-rank tests did not record difference between patients who received mCH alone and those who received mCH + RT in term of PFS. Conversely, a much longer survival was recorded in the second group of patients [mCH *vs*. mCH+RT: 12.1 +/-2.5 (95%CI 3.35-8.6) *vs*. 22.12 +/-4.3 (95%CI 11.9-26.1) months; *P* = 0.015] (Figure 1A, 1B). Kaplan Meyer curves and log-rank tests on the group of patients who received RT did not disclose significant correlations between either PFS or OS and with: a) sex, b) grading, c) ECOG performance status score (1 < *vs*. ≥1) and type response (PR *vs* SD) to the mCH (data not shown).

### Immunomodulation and survival

**Figure 2:**
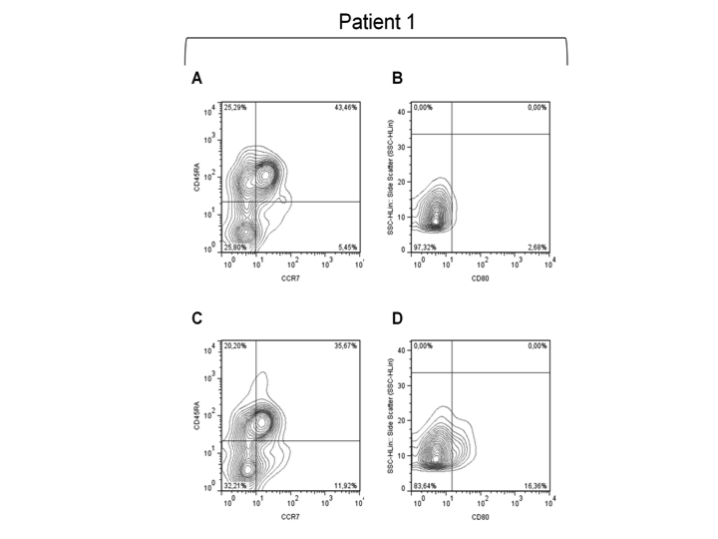
**Immuno cytofluorimetric analysis performed on the PBMCs of a representative patient**. An immuno cytofluorimetric analysis performed on the PBMCs of a representative patient and showing baseline and post-treatment expression of activated DCs (CD11b+CD83+CD80+) and TCMs (CD3+CD8+CD45A-CCR7+). This analysis shows a significant increase in both cell population after four mPEBev courses. **A**. and **C**. represent, respectively, pre- and post-treatment expression of TCM; **B**. and **D**. represent, respectively, pre- and post-treatment expression of CD80.

We then investigated whether the longer survival recorded in patients who received RT and mCH could have some correlation with the immunological effects induced by the mPEBev regimen in NSCLC patients and described in a previous report [41]. Our analysis revealed a much longer survival in those patients who received RT + mCH and presented a mPEBev-related increase in activated DCs (CD3-CD11b+CD15-CD83+CD80+) [FBV ≤1 *vs* > 1: 4+/-5.389 (95%CI,0-14.56) *vs* 56+/-23.05 (95%CI,10.8-101.2) months; p:0.049)] and peripheral TCMs [FBV ≤1 *vs* > 1: 8+/-5.96 (95%CI,0-19.68) *vs* 31+/-12.3 (95%CI,6.94-55.1) months; p:0.046] (Figure 1C, 1D and Figure 2). We were instead unable to demonstrate the same survival correlation in patients who received the mCH alone with the treatment-related increase in these blood cell populations [DC, FBV ≤ 1 *vs* > 1; 14.6+/-5.618 (95%CI 0-12.046) *vs* 6+/-1.35 (95%CI1.6-10.325) months, *P* = 0.800; TCMs FV ≤1 *vs* > 1: 17.7+/-1063 (95%CI 3.54-8.46) *vs* 10.7+/-3.84 (95%CI,1.4 4.55); *P* = 0.559], (Figure 1E, 1F). Our statistical analysis failed to demonstrate any other survival correlation with baseline and mCH-related changes in the serum levels of proangiogenic factors (VEGFA, Angiopoietin 2 and Follistatin), cytokines [Interferon (IFN)0263, IL4 and IL17A] and Tregs, NKs or MDSC (data not shown).

## DISCUSSION

Our retrospective analysis, carried out on advanced NSCLC patients who completed four mPEBev regimen courses and received concomitant or subsequential palliative radiotherapy, revealed a 78% disease control rate (CR+PR+SD) with an OS over 23 months and a 66% one-year survival rate. These patients presented a much longer OS if compared with a homologous group of patients enrolled in the same BEVA2007 trial and who underwent the same mCH regimen with no progression along the treatment, who did not receive RT. Our analysis showed that the OS of patients who received mCH + RT did not correlate with RT modality, dosage or site of irradiation. Interestingly, the two populations of patients who received mCH or mCH + RT did not show statistical difference in term of PFS, a fact that suggests a systemic therapeutic effects in the latter group of patients which results in a prolonged survival.

Even though significant bias exists, due to the small sample, and the retrospective nature of the study, these results appear intriguing. In fact, the most promising systemic treatments for inoperable NSCLC patients present a median OS barely longer than 12-14 months [3–5]. Additionally, no information, concerning the putative ability of palliative RT to prolong the survival in patients who receive standard chemotherapy +/- bevacizumab is currently available. We have hypothesized that our metronomic CH regimen may help RT to elicit an immune-mediated response, with potential antitumor activity as described in the literature [32, 33, 42]. This hypothesis is strongly supported by the finding that the survival of patients who received mCH + RT is correlated with the mPEBev-dependent increase in activated DCs and TCMs. In line with these results, preclinical findings suggest that RT-induced tumor cell death and peri-tumoral inflammation modulate the immune-system's ability to activate antigen presenting cells-mediated cross-priming and additional immune-effects (immunogenic cell death). Exposure of tumor cells to ionizing radiation can result in immunogenic cell death, whereby upregulation or release of new tumoral antigens and of Damage-Associated-Molecular-Patters (DAMPs), Heat-Shock-Proteins and High-Mobility-Group-Box-1 (HMGB1), recognized by the toll like receptor-4 (TLR-4) on the surface of DCs, promotes both maturation and activity of DCs [42, 43]. Therefore, the release of DAMPs associated with RT-induced cancer cell death occurs in a dose-dependent fashion and has been shown to recruit and stimulate DCs to uptake tumor antigens and cross-present them to naïve T cells thus initiating anti tumor immune responses. RT can also facilitate the recruitment of effector T-cells to the tumor by inducing the secretion of CXC motif chemokine ligand CXCL9, CXCL10 and CXCL16 by tumor cells [1, 2]. RT is able to induce upregulation of major histocompatibility complex class I molecules, FAS/CD95, and stress-induced natural killer group 2D-ligands on tumor cells by enhancing recognition and killing of tumor cells by CTls [1, 3, 4]. Thus, all radio-induced immunological effects convert the irradiated tumor cells into an “*in situ* vaccine”, resulting in immunogenic cell death and activation of a systemic antitumor immune response [42–44]. In this context, it has been shown that the sequential or concomitant combination of RT with anticancer vaccines elicits a very efficient multi-antigen T cell antitumor response in mouse models that leads to longer survival compared with either vaccine-therapy or RT alone [6, 45].

On these basis, we can speculate the existence of an additive antitumor effect between mPEBev mCH and RT potentially related to clear immune-biological effects of both treatment modality. The mPEBev-related increase in the percentage of activated DCs could in fact, empower the *cross-priming* of antigen released by the irradiated tumor tissue, with a consequential amplified anti-tumor T cell response. In this contest, TCMs represent an effector-T-cell subset with long term memory and high tumor killing activity, which are able to achieve distant lymph-nodes and tumor sites due to the expression of chemokine receptors (CCR)-7 on their surface [46, 47]. However, despite of the immune-stimulatory effects of both mPEBev regimen and RT, cancer recurrence and/or progression occurs in the majority of patients due to immune-escape mechanisms in the long term. In this context PD-1/PDL1 immune-check point blockade with mAbs could be a promising therapeutic salvage tool in these patients. In general, the main limitation of these immune-checkpoint blocking agents is represented by an inefficient tumor specific CTL response and by a low CTL-tumor infiltration rate which precedes the immune-checkpoint blockade. At this purpose a rationale combination of PD-1/PDL1 blockade with treatments, like chemotherapy, RT or cancer vaccines, able to elicit an efficient CTL response that could produce more than additive therapeutic results in these patient has already been proposed. In this context, Deng et al. showed that RT-mediated inflammation results in IFNγ release thus increasing PDL-1 expression on cancer cells, MDSCs, and M2-macrophages in tumor microenvironment [30] and also reported that the combination of RT and mAbs to PDL- 1 enhances the frequency of the abscopal events and the antitumor activity of both treatment modalities [30].

In conclusion, although with significant limitation, our results suggest that RT delivered on a palliative setting prolongs the survival of advanced NSCLC patients who have received the mPEBev regimen presumably by igniting an immunological effect. This effects seems to be correlated to the development of antigen presenting cells like DCs and consequently to the expansion of activated effector T cells with antitumor activity. On these basis, this sequential module of treatment which involves mPEBev regimen + stereotactic RT deserves to be investigated in further prospective trials in NSCLC patients possibly in sequential combination with immune-checkpoint blockade.

## PATIENTS AND METHODS

### Study design

The study protocol code #BEVA2007 was a two step phase I/II clinical trial, performed in accordance to the good clinical practice guidelines and was approved by the Bioethics Committee of the University of Siena as described in previous reports [38–41]. Moreover, informed consent was obtained from each subject or subject's guardian. The first step of the study included 25 patients, who were sub-divided in five cohorts receiving escalating dosage of bevacizumab. Cohort 1 received mPE chemotherapy alone, while cohort 2, 3, 4 and 5 received bevacizumab every three weeks, at the dosage of 2.5, 5, 7.5 and 10 mg/kg every three weeks [38]. Bevacizumab dosage for the second step (Phase II trial) was extrapolated by the results of the first step that identified 5 mg/kg as the most effective biological dose (MEBD) in combination with the mPEBev regimen and 7.5 mg/kg as its maximum tolerated dose (MTD). Sixty-nine patients signed a written informed consent and were enrolled in the second part of the study [39–41]. All the cases were discussed in a multidisciplinary tumor board, including a dedicated surgeon, medical oncologist, radiation oncologist, radiologist, and pathologist. The inclusion criteria were: histological diagnosis of mNSCLC, allowing squamous cell carcinomas not considered at risk of bleeding, performance status (ECOG) from 0 to 2, normal renal and hepatic function, WBC count more than 2,500/mm3, hemoglobin more than 9 g/dl, platelet cell count more than 90,000/mm3, normal cardiac function, and advanced stage (IIIB/IV) of disease. The exclusion criteria were: Central tumors with high risk of bleeding (excavated with bulky necrosis and infiltration of large arterial and venous structures) for bevacizumab use, a history of other severe cardiovascular disease, arrhythmia, second malignant tumors, signs of active infections.

### Treatment schedule

Sixty-nine patients, therefore, received iv. cisplatin (30 mg/sqm) on days 1-3 and daily oral etoposide (50 mg) on from day 1 to day 15 and bevacizumab (5 mg/kg) on the day 3 every three weeks, for a maximum of 4 consecutive courses [38–40]. Palliative radiotherapy was allowed with palliative intent on quality of-life threatening lesions.

### Radiation therapy treatment

RT was delivered on a case by case basis, as a palliative treatment. Forty-five of these patients received radiation therapy, either as RT to the bones (19 patients), WBRT (18 patients) and SRS (3 patients), palliative thoracic irradiation (7 patients). RT was delivered with a Linear Accelerator (6MV-15MV photon) as a 3D-conformational radiation therapy (3D-CRT) on that. The target volume was identified by diagnostic CT scan. RT dosage was prescribed on a case by case basis, according to the Clinician choice. CT simulation was performed with a 5 mm slicing, 120KV, 10 Index Noise, Range 100-440 mA, spiral 16 slices CT Scanner.

### Biological analysis and blood sampling

Peripheral blood samples (10 ml) were withdrawn at baseline and one hour before any treatment cycle of chemotherapy for either serum and PBMC isolation. Serum derived from standard peripheral blood centrifugation and PBMCs obtained by Ficoll-Hypaque (Celbio S.P.A., Italy) gradient separation medium form heparinized blood samples were immediately frozen and stored as described in previous studies [38–40]. Lymphocytes, platelets, neutrophils, monocytes were evaluated by hemocytometric cell counts, while their feature was evaluated by microscope analysis. Flow cytometry was perfomed on patients’ PBMCs by carrying out standard multicolor immuno-cytofluorimetric analysis [41] with conjugated anti-CD3, CD4, CD8, CD27, CD62L, CD19, CD16, CD56, CD25, CD80, CD83, FoxP3, CCR7, CD45Ra, CD11b, CD11c, CD14, CD15 mAbs all purchased by eBioscience, USA.

### BioPlex assay

Blood samples were collected from a peripheral vein at baseline and just before each treatment and kept on ice. Serum was collected by centrifugation (3,000 rpm for 10 min at 4°C), aliquoted, and stored at −80°C until analyzed. A multiplex biometric ELISA-based immunoassay, containing dyed microspheres conjugated with a monoclonal antibody specific for a target protein was used according to the manufacturer's instructions (Bioplex, Bio-Rad Lab., Inc., Hercules, CA, USA). Soluble molecules were measured using either commercially available kits or customized kits for the evaluation of the following cytokines: Interleukin(IL)4, IL8, IL10, IL12/A, IL17/A, IFN0263, Tumor necrosis factor (TNF)α, VEGF, Granulocyte-Colony Stimulating Factor (GCSF), angiopoietin-2 as described in previous papers [38–41]. Serum levels of all proteins were determined using a Bio-Plex array reader (Luminex, Austin, TX) that quantifies multiplex immunoassays in a 96-well plate with very small fluid volumes. The analyte concentration was calculated using a standard curve, with software provided by the manufacturer (Bio-Plex Manager Software).

### Statistical analysis

The between-mean differences were statistically analyzed using Stat View statistical software (Abacus Concepts, Berkeley, CA). The results were expressed as the mean +/- standard deviation (SD) of 4 determinations made in three different experiments, and the differences determined using the 2-tail Student's *t*-test for paired samples. In order to perform a survival analysis we divided the patients into two subgroups with low (A) and high (B) score, according to their respective median value of each specific marker or treatment related level change expressed as fold change to baseline value. Descriptive statistic by Kaplan Meier's method and Log-Rank test were used to evaluate PFS, and OS and correlate them with patients’ associated variables. All analyses were performed by SPSS statistical package, version 17.0. A *p*-value of 0.05 or less was considered statistically significant. In the comparative analysis, patients who showed a rapid disease progression or severe adverse events or death during the induction treatment with the mPEBev regimen and did not complete at least three treatment courses were excluded from the retrospective study.

## Abbreviations

RT: radiotherapy
mCH: metronomic chemotherapy
mPEBev: metronomic dose-fractioned cisplatin, oral etoposide and bevacizumab
mNSCLC: metastatic non-small-cell-lung cancer
EGFR: epidermal growth factor receptor
EML-ALK: echinoderm microtubule-associated protein-like 4 and anaplastic lymphoma kinase fusion protein
VEGF: vasculo-endothelial-growth-factor
OS: overall survival
PFS: progression-free survival
mAbs: monoclonal antibodies
PD-1: programmed cell death receptor-1
PDL-1: PD-1 ligand-1
CTLs: cytotoxic-T-cells
IMRT/VMAT: intensity/volumetric modulated arc therapy
IGRT: image-guided RT
Tregs: regulatory-T-cells
MDSCs: myeloid-derivative-suppressor-cells
TCMs: central-memory-T-cells
PBMCs: peripheral blood mononuclear cells
DC: dendritic cells
ECOG: eastern cooperative oncology group performance status
WBRT: whole brain radiotherapy
SRS: stereotactic radiosurgery
Gy: gray
PR: partial response
SD: stable disease
FBV: fold to baseline value
VEGFA: vascular endothelial growth factor A
IFN0263: Interferon 0263
DAMPs: Damage-Associated-Molecular-Patters
HMGB1: high-mobility-group-box-1
TLR-4: toll like receptor-4
CCR-7: chemokine receptors
MEBD: most effective biological dose
MTD: maximum tolerated dose
WBC: white blood cell
3D-CRT: 3D-conformational radiation therapy
TNFα: tumor necrosis factor α
GCSF: granulocyte-colony stimulating factor
